# Multilevel regulation of Wnt signaling by Zic2 in colon cancer due to mutation of β-catenin

**DOI:** 10.1038/s41419-021-03863-w

**Published:** 2021-06-07

**Authors:** Zhengshui Xu, Jianbao Zheng, Zilu Chen, Jing Guo, Xiaopeng Li, Xingjie Wang, Chao Qu, Liyue Yuan, Chen Cheng, Xuejun Sun, Junhui Yu

**Affiliations:** 1 Department of General Surgery, First Affiliated Hospital of Xi’an Jiaotong University, Xi’an, 710061 Shaanxi Province PR China; 2 Department of Endocrinology, First Affiliated Hospital of Xi’an Jiaotong University, Xi’an, 710061 Shaanxi Province PR China

**Keywords:** Colon cancer, Cell growth

## Abstract

Zinc-finger of the cerebellum 2 (Zic2) is widely implicated in cancers, but the role of Zic2 in tumorigenesis is bilateral. A recent study indicated that Zic2 could render colon cancer cells more resistant to low glucose-induced apoptosis. However, the functional roles of Zic2 in colon cancer and the underlying molecular mechanism remain elusive. Herein, we demonstrated that Zic2 was highly expressed in colon cancer tissues and correlated with poor survival. Knockdown of Zic2 inhibited colon cancer cell growth, arrested the cell cycle transition from G0/G1 to S phase, and suppressed tumor sphere formation in vitro; in addition, silencing Zic2 retarded xenograft tumor formation in vivo. Consistently, ectopic expression of Zic2 had the opposite effects. Mechanistically, Zic2 executed its oncogenic role in colon cancer by enhancing Wnt/β-catenin signaling. Zic2 directly binds to the promoter of Axin2 and transcriptionally represses Axin2 expression and subsequently promotes the accumulation and nuclear translocation of β-catenin. Meanwhile, Zic2 could activate Wnt signaling by interacting with β-catenin. Intriguingly, in HCT116 cells with intrinsic Ser45 mutation of β-catenin, which blocks the degradation-related phosphorylation of β-catenin by CK1, modified Zic2 expression did not affect the protein level of β-catenin. Altogether, our findings uncover a novel multilevel mechanism for the oncogenic activity of Zic2 in colon cancer and suggest Zic2 as a potential therapeutic target for colon cancer patients.

## Background

Colorectal cancer (CRC) is the second most prevalent cancer and the fourth leading cause of cancer-related lethality globally^1^. Approximately 90% of CRCs have abnormal Wnt signaling activation, which plays important role in cancer stem cell (CSC) biology, tumorigenesis, and metastasis in CRC^2–5^. Nuclear translocation of β-catenin is considered the hallmark of the canonical Wnt pathway^2,4,6^. The disruption of the β-catenin destruction complex, consisting of CK1^7,8^, GSK-3β^9,10^, Axin^7,11^, and APC^10,12^, results in the activation of Wnt signaling. Among the β-catenin destruction complex, AXIN2, playing crucial role in Wnt/β-catenin signaling pathway, participates in regulating cell proliferation, apoptosis, migration, and other important functions in the development of various cancers^13^. It is generally accepted that CK1, by binding to Axin2, initiates β-catenin degradation by phosphorylation at Ser45; subsequently, GSK-3β catalyzes the phosphorylation of β-catenin at more N-terminal residues (Ser33, Ser37, and Thr41)^5–7,14^, while APC can intensify the affinity of phosphorylases for β-catenin to accelerate β-catenin degradation in the cytoplasm via the ubiquitin-proteasome^2,6^. Dephosphorylated β-catenin accumulated in the cytoplasm can translocate into the nucleus and form complexes with member(s) of the TCF/LEF family to initiate transcription of downstream target genes, such as cyclin D1, CD44, c-Myc, and Lgr5^15–18^. Mutations in APC^5,19^, β-catenin^2,14^, and Axin1^17^ in CRC block the destruction complex’s affinity for β-catenin, which triggers aberrant accumulation of β-catenin. Among colon cancer cell lines, HCT116 has a special three-base deletion in the β-catenin encoding gene, *CTNNB1*, causing an in-frame deletion at Ser45^20^.

Zinc-finger protein of the cerebellum 2 (Zic2), homologous to the Drosophila protein odd-paired, belongs to the Zic family of proteins, which contain highly conserved C2H2 zinc-finger motifs^21–23^. As a transcription factor, Zic2 plays important role in cerebral formation in both animals and humans^24,25^. Mutations in Zic2 cause spina bifida and axial and limb bone malformation^23^. Likewise, Zic2 functions in tumorigenesis^26^, cancer stem cell renewal^21^, proliferation^27–31^, invasion, and metastasis^28,30,32^. However, the functions and mechanisms of Zic2 in various cancers are extraordinarily complicated and remain unclear^29^. In CRC, only one Zic2-related study has been published, in which Zhao et al. reported that Zic2 can render colon cancer cells more resistant to low-glucose-induced apoptosis^31^. However, how Zic2 regulates CRC tumorigenesis and progression remains elusive.

## Materials and methods

### Clinical samples

A tissue microarray of 180 pairs of primary CRC tissues was purchased from Shaanxi Kexin Biotechnology Co., Ltd (cohort I). Four hundred and three colon cancer tissue samples and paired normal colon tissue samples were randomly selected from CRC patients who had not received radiotherapy or chemotherapy before excision between January 2011 and December 2016 (cohort II). All the patients underwent surgery at the First Affiliated Hospital of Xi’an Jiaotong University. Informed consent forms were signed by all patients. Our study protocol was approved by the Ethics Committee of the First Affiliated Hospital of Xi’an Jiaotong University.

### Lentiviral vectors and transfection

Lentiviral vectors with Zic2 shRNA or Zic2 overexpression were purchased from GeneChem Co., Ltd. (Shanghai, China). The target short hairpin RNA (shRNA) sequences were presented in Supplementary Table 3. Lentiviral infection referred to the manufacturer’s protocol.

### Immunohistochemistry (IHC)

For IHC, the staining procedure was performed using the standard avidin-biotin complex method. The extent of positively stained cells was scored as previously described^33^. The Zic2-stained (Avia Systems Biology, San Diego, CA, USA) sections were divided into two groups (High and Low) based on scores of the staining between colon cancer tissues and adjacent colon normal tissues. Two pathologists evaluated all the specimens in a blinded manner.

### RNA isolation and real-time PCR

Total RNA was isolated from cells using TRIzol reagent (Invitrogen, Carlsbad, CA, USA). Complementary DNA (ctDNA) was synthesized by the PrimeScript RT Reagent Kit (TaKaRa, Osaka, Japan). Real-time PCR was conducted on an IQ5 instrument (Bio-Rad, CA, USA) using SYBR Green fluorescence signal detection assays (TaKaRa, Osaka, Japan) with primers list in Supplementary Table 4. The specific mRNA expression level was quantified by using the 2 − ΔΔCT method^33^.

### Luciferase reporter assay

For promoter analyses, a fragment of the Axin2 5′-flanking sequence (from −912 to +207 bp) and other truncated fragments were cloned into the pGL3.0 Basic Vector (Promega, Madison, WI, USA) to generate a Axin2 full promoter reporter construct and the truncated ones (Supplementary Table 1). The plasmids containing firefly luciferase reporters of PTEN promoter and the truncated ones and the pTK-RL plasmids were co-transfected into cells. The detailed protocol was carried out as described previously^33^.

### Quantitative chromatin immunoprecipitation (qChIP)

The qChIP assay was conducted using the EZ-ChIP Kit (Millipore, Bedford, MA, USA) according to the method of the manufacturer’s instructions^33^. 5 μg anti-Zic2 antibody and 1 μg IgG negative control antibody were used to precipitate the chromatin-protein mixture. Finally, the target fragment or endogenous non-coding region fragment were amplified with specific primers (Supplementary Table 1) by using real-time PCR.

### Cell cultures

DLD-1, HCT116, and SW480 cells (Shanghai Institute of Cell Biology, Chinese Academy of Sciences) were authenticated by STR analysis (GeneChem Co., Ltd., Shanghai, China). Cell lines were maintained in DMEM and McCoy’s 5A medium (HyClone, Logan, UT, USA) supplemented with 10% FBS (Gibco BRL, Carlsbad, CA, USA) and 100 units/ml of penicillin and streptomycin at 37 °C in a humidified 5% CO_2_ atmosphere.

### Immunohistochemistry (IHC)

For IHC, the staining procedure was performed using the standard avidin–biotin complex method. The extent of positively stained cells was scored as previously described^33^. The Zic2-stained (Avia Systems Biology, San Diego, CA, USA) sections were divided into two groups (High and Low) based on scores of the staining between colon cancer tissues and adjacent colon normal tissues. Two pathologists evaluated all the specimens in a blinded manner.

### Tumor spheres

Cells were counted and plated in SFM [DMEM/F12 medium (Thermo Scientific, Mulgrave, Australia), 1% B27 supplement without vitamin A (Thermo Scientific), 20 ng/ml EGF (Thermo Scientific), and 20 ng/ml FGF (R&D Systems, Minneapolis, MN)] in ultralow attachment six-well plates (Corning, Tewksbury, MA) at a total of 1.5 × 10^3^ cells per well, and cultured for up to 2 weeks to allow for spheroid growth. The medium was refreshed every 3 days.

### Cell counting kit-8 (CCK8) assay

Cells were incubated at 5% CO_2_ and 37 °C on 96-well plates (100 μL/well). Ten microliters of CCK-8 reagent (Dojindo, Kyushu, Japan) was added to each well after 24, 48, 72, and 96 h, respectively. OD450 values were determined by a microplate reader.

### Colony formation assay

Cells were plated into 60-mm plates and routinely cultured for 14 days. The cells were subsequently fixed with methyl alcohol for 15 min and stained with 0.1% crystal violet.

After staining, the visible colonies were counted.

### Cell cycle assay

Cells were harvested and fixed in 75% cold ethanol and stored at 4 °C overnight. The next day cells were centrifuged at 1500 rpm for 5 min. After treatment with RNase A at 37 °C for 30 min, cell pellets were further washed with PBS and stained with PI (10 μg/100 μl) for 10 min in the dark. The cell cycle was assessed with flow cytometry (Beckman Coulter).

### Nude mouse xenograft assay

The use of all animals in this study was approved by the Institutional Animal Care and Use Committee of the First Affiliated Hospital of Xi’an Jiaotong University. Tumor cells (5 × 10^6^) in logarithmic phase were sub-cutaneously injected into the right flanks of 5-week-old female BALB/c-nude mice (Shanghai SLAC Laboratory Animal Co. Ltd., Shanghai, China). After 1 week post-injection, the length (*a*) and width (*b*) of the tumor were monitored using callipers every 3 days. The tumor volume (*V*) was calculated as follows: *V* = *ab*^2^/2. Three weeks later, the mice were sacrificed and the xenograft tumors were measured.

### Protein extraction and western blotting

Cells were lysed using RIPA buffer (Heart, Xian, China). Cell lysates containing 30 μg of total protein were then subjected to SDS–PAGE (Beyotime, Shanghai, China) and then transferred to PVDF membranes (Millipore, Billerica, MA, USA). The membranes were incubated with primary antibodies overnight at 4 °C (anti-Zic2, Axin2, APC, active-β-Catenin(ser45), β-Catenin, Flag, and c-Myc, 1:1000 dilution; Cyclin D1 and GAPDH, 1:5000). The membrane was then washed six times with TBST buffer for 5 min each and incubated with a horseradish peroxidase-conjugated secondary antibody at room temperature for 1 h. Chemiluminescent HRP substrate (Millipore, Billerica, MA, USA) was added to visualize the protein bands. The antibodies against GAPDH were purchased from Santa Cruz (Dallas, TX, USA), the antibodies against Zic2, Cyclin D1, CD44, Axin2, and APC were purchased from Abcam (Cambridge, MA). The antibodies against active-β-Catenin(ser45), β-Catenin, and GSK-3β were purchased from Cell Signaling Technology (Danvers, MA, USA), and the antibodies against Flag was purchased from Proteintech (Rosemont, USA). Nuclear extract was prepared with the protocol in the Nuclear Extraction Kit (Abcam, Cambridge, MA, USA).

### RNA-seq analysis

The sequencing data was filtered with SOAPnuke (v1.5.2)^34^ by (1) Removing reads containing sequencing adapter; (2) Removing reads whose low-quality base ratio (base quality less than or equal to 5) is more than 20%; (3) Removing reads whose unknown base (‘N’ base) ratio is more than 5%, afterward clean reads were obtained and stored in FASTQ format. The clean reads were mapped to the reference genome using HISAT2 (v2.0.4)^35^. Bowtie2 (v2.2.5)^36^ was applied to align the clean reads to the reference coding gene set, then expression level of gene was calculated by RSEM (v1.2.12)^37^. The heatmap was drawn by pheatmap (v1.0.8)^38^ according to the gene expression in different samples. Essentially, differential expression analysis was performed using the DESeq2(v1.4.5)^39^ with *Q* value ≤0.05. To take insight to the change of phenotype, GO (http://www.geneontology.org/) analysis of annotated different expressed gene was performed by Phyper (https://en.wikipedia.org/wiki/Hypergeometric_distribution) based on Hypergeometric test. The significant levels of terms and pathways were corrected by *Q* value with a rigorous threshold (*Q* value ≤ 0.05) by Bonferroni^40^.

### Co-immunoprecipitation (Co-IP) assays

Cells were lysed in Triton X-100 lysis buffer [NaCl (150 mM), NP-40 (0.5%), Tris-HCl (pH = 8.0), glycerol (20 mM, 20%)] containing phosphatase inhibitors and protease inhibitor (Roche, NJ, USA), then the proteins extract were centrifugated at 12,000 × *g* for 20 min. Thermo Scientific Pierce Co-IP kit (Thermo Fisher Scientific) was used for Co-IP experiments. At last, obtained proteins were resuspended in 5× SDS sample loading buffer, heated to 100 °C for 8 min, and tested by 10% SDS–polyacrylamide gel electrophoresis (PAGE).

### Immunofluorescence (IF)

The cells were washed three times with PBS for 10 min each, fixed with 4% paraformaldehyde for 20 min, and permeabilized with 0.2% Triton X-100 for 10 min. After blocking with 5% bovine serum albumin (BSA) for 30 min at room temperature, the cells were incubated at 4 °C overnight with primary antibodies against Zic2 (1:100 dilution). The dishes were washed three times with PBS for 10 min each and then incubated with Alexa Fluor 594-conjugated secondary antibodies (1:400 dilution, Invitrogen, Carlsbad, CA, USA) for 1 h at room temperature. The nuclei were stained with DAPI (10 mg/ml) for 10 min. The samples were examined via microscopy (Leica Microsystems, Heidelberg, Germany) to analyze the expression and nuclear location of Zic2.

### Statistical analysis

All data are presented as the mean ± standard deviation (SD). The chi-square test or ANOVA was used to analyze the differences among groups. Correlations were analyzed using Pearson linear-regression analysis. OS and RFS rates were plotted using the Kaplan–Meier method and compared with log-rank test. Multivariate statistical analysis was performed using a Cox regression model. All statistical analyses were performed using SPSS 22.0 software (SPSS Inc., Chicago, IL, USA). *P* < 0.05 was defined as statistically significant.

## Results

### Zic2 is upregulated in colon cancer samples, and high Zic2 expression correlates with unfavorable survival of colon cancer patients

**Fig. 1:**
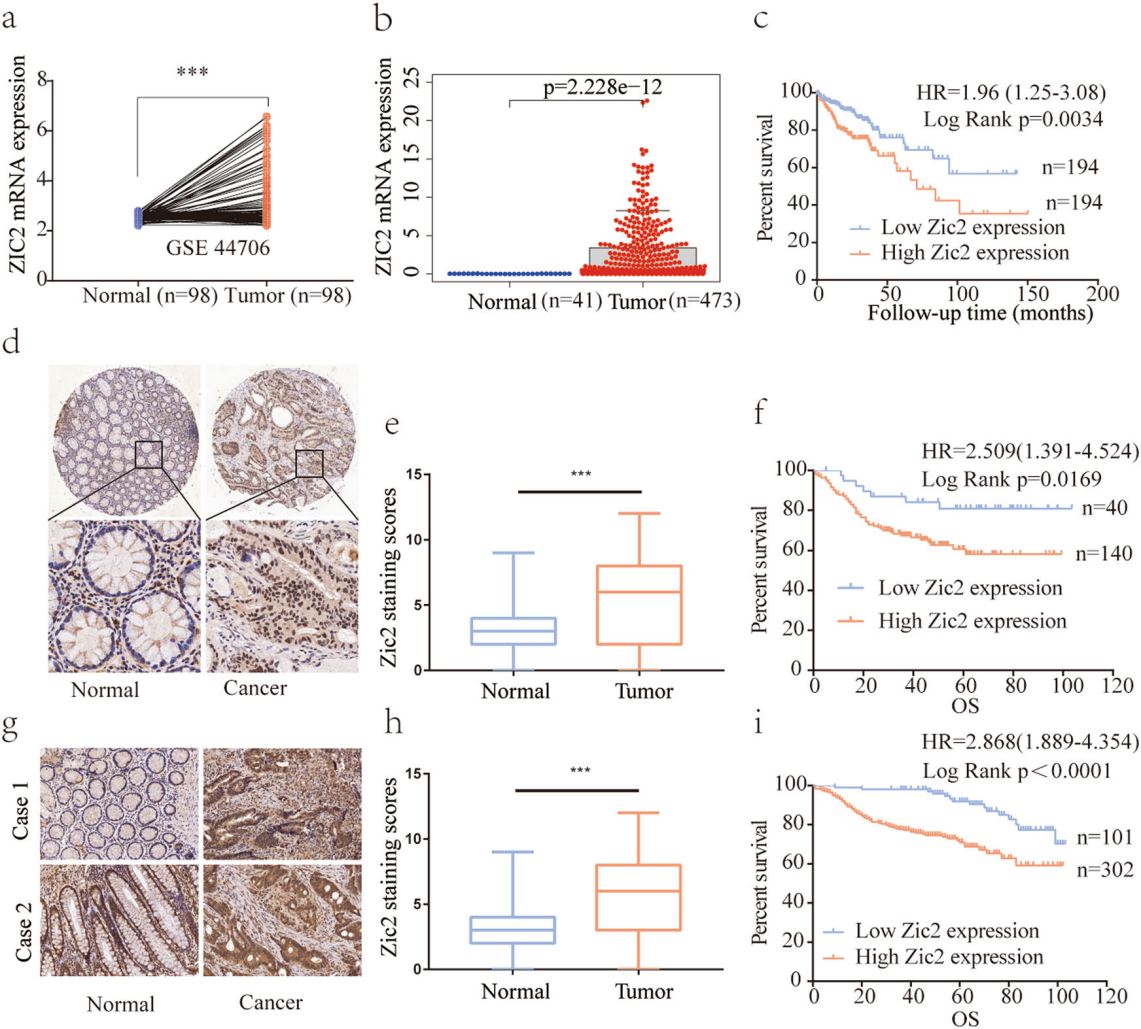
The expression of Zic2 in colon cancer tissue samples and normal tissue samples. **a** Zic2 mRNA expression of 98 colon cancer tissue samples and paired NC tissue samples in GSE 44706 mRNA microarray. **b** Zic2 transcription expression of 473 colon cancer tissue samples and 41 NC tissue samples in TCGA database. **c** Data in TCGA database showed the overall survival of the two groups of patients with high (*n* = 194, red line) or low (*n* = 194, blue line) Zic2 expression in colon cancer tissues. **d**, **g** Representative images of the IHC staining of Zic2 in two independent cohorts (Cohort I (**d**), *N* = 180; Cohort II (**g**), *N* = 403). **e, h** The staining scores of the Zic2 expression in two independent cohorts. **f**, **i** Kaplan–Meier representation of the overall survival between the two groups of patients with high (red line) and low (blue line) Zic2 expression in two independent cohorts. All data are the mean ± SD of three independent experiments. ***P < 0.0001.

To explore the role of Zic2 in colon cancer, we first analyzed the expression of Zic2 between colon cancer and normal colon tissues by bioinformatic analysis. The results showed that Zic2 transcript levels were upregulated in colon cancer compared with normal colon tissues in the GSE44706 mRNA microarray dataset (Fig. 1a) and TCGA RNA-sequencing data (Fig. 1b). Zic2 might be a potential prognostic marker in colon cancer because higher Zic2 transcript levels were strongly correlated with poorer overall survival in TCGA data (Fig. 1c).

To confirm the role of Zic2 proteins in colon cancer, we performed immunohistochemical (IHC) staining of 180 colon cancer samples via a tissue microarray (Fig. 1d–f, cohort I) and found a markedly increased protein level of Zic2 in colon cancer tissues compared with adjacent colon normal tissues (*P* < 0.0001, Fig. 1d, e). Zic2 protein was mainly localized in the nucleus of cells in colon cancer tissues (Fig. 1d) and colon cancer cell lines (Supplementary Fig. 1a). High expression of Zic2 protein was correlated with poorer overall survival (OS) than low expression of Zic2 in colon cancer patients (Fig. 1f). To further validate the role of Zic2 in colon cancer, we performed IHC staining using another independent cohort of 403 colon cancer patient samples (Fig. 1g–i, cohort II). The results were consistent with those observed in cohort I. Moreover, higher Zic2 protein expression was significantly associated with a poorer tumor phenotype, including larger tumor size, a higher rate of stage T3/T4 tumors, and higher AJCC stage (Supplementary Table 1). By multivariate analysis, we found that Zic2 was an independent prognostic factor for poor OS in colon cancer patients (Supplementary Table 2). Altogether, these data indicate that Zic2 is upregulated in colon cancer samples and that high Zic2 expression correlates with unfavorable survival of colon cancer patients.

### Zic2 mediates colon cancer cell proliferation by inducing a transition from G0/G1 to S phase in vitro

The expression of Zic2 was measured in six established colon cancer cell lines and one human colonic epithelial cell line (HCoEpiC) by western blotting and qRT-PCR analysis (Supplementary Fig. 1b, c). We found that Zic2 transcript levels were higher in all six colon cancer cell lines than in HCoEpiC. To further gain insight into the impact of Zic2 in colon cancer, a series of in vitro and in vivo experiments were performed in colon cancer cells with silenced or overexpressed Zic2. Silencing of Zic2 in DLD-1 and HCT116 cells and ectopic expression of Zic2 in DLD, HCT116, and SW480 cells were validated by western blotting and qRT-PCR analysis (Supplementary Fig. 1d–j).

**Fig. 2:**
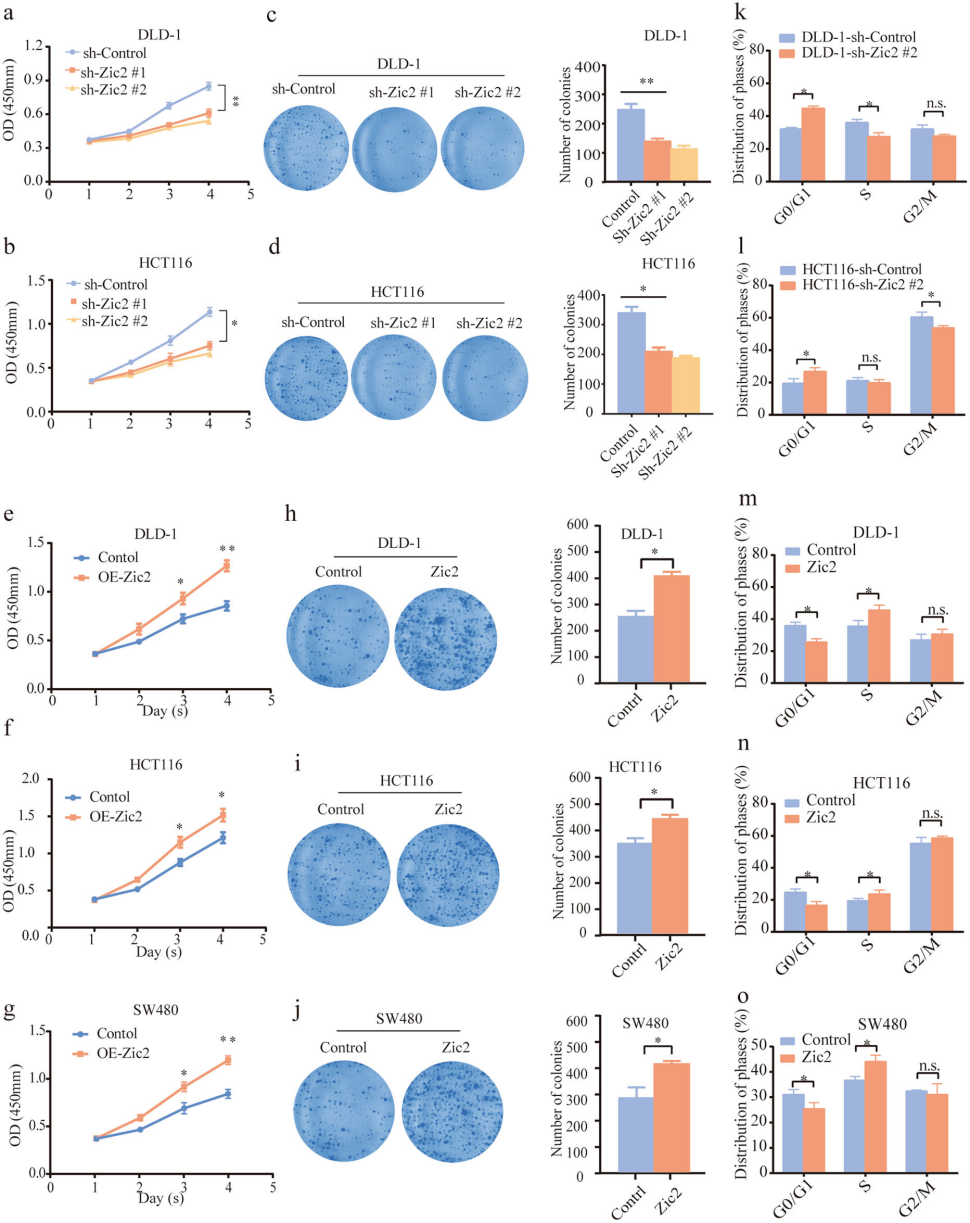
Zic2 mediates colon cancer proliferation in vitro. **a**, **b** CCK8 proliferation assay in DLD-1 (**a**) or HCT116 (**b**) cells transfected with sh-Zic2 or vector. **c**, **d** Colony formation in DLD-1 (**c**) or HCT116 (**d**) cells transfected with sh-Zic2 or vector. **e**–**g** CCK8 proliferation assay in DLD-1 (**e**), HCT116 (**f**) or SW480 (**g**) cells transfected with Zic2 or vector. **h**–**j** Colony formation in DLD-1 (**e**), HCT116 (**f**) and SW480 (**g**) cells transfected with sh-Zic2 or vector. **k**, **l** Comparison of the cell cycle distribution between Zic2-knockdown cells [DLD-1 (**k**) and HCT116 (**l**)] and vectors. **m**–**o** Comparison of the cell cycle distribution between Zic2-overexpressing cells [DLD-1 (**m**), HCT116 (**n**) and SW480 (**o**)] and vectors. All data are presented as the mean ± SD from three independent experiments. *P < 0.05, **P < 0.01.

To investigate the function of Zic2 in vitro, CCK-8 and colony formation assays were performed. As shown in Fig. 2a–d, knockdown of Zic2 repressed the viability and colony formation of DLD-1 and HCT116 cells. Conversely, DLD, HCT116, and SW480 cells with Zic2 overexpression exhibited increased viability and colony formation (Fig. 2e–j). We then attempted to reveal the potential mechanism by which Zic2 mediates colon cancer cell proliferation by analyzing the cell cycle and apoptosis. The results showed that depletion of Zic2 arrested DLD-1 and HCT116 cells in the G0/G1 phase (Fig. 2k, l, Supplementary Fig. 2a–d). Conversely, ectopic expression of Zic2 induced a transition from G0/G1 to S phase in DLD-1, HCT116, and SW480 cells (Fig. 2m–o, Supplementary Fig. 2e–k). Intriguingly, modulation of Zic2 expression did not affect the apoptosis rate in colon cancer cells (Supplementary Fig. 3). Collectively, these data indicate that Zic2 promotes colon cancer proliferation by inducing a transition from G0/G1 to S phase rather than by impacting apoptosis.

### Zic2 correlates with colon CSC properties

**Fig. 3:**
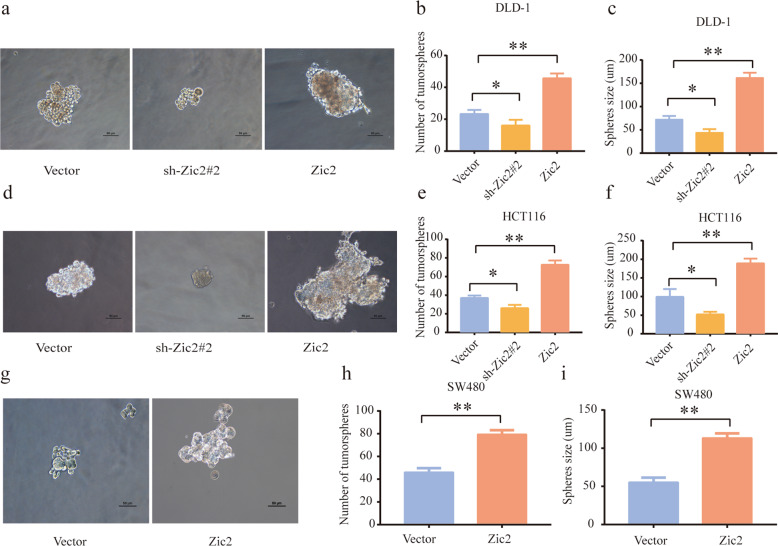
Zic2 expression positively correlates with colon CSCs property. **a**, **d**, **g** Representative images of tumorsphere from cell lines with different expression levels of Zic2**. b**, **e**, **h** Sphere number in cell lines with different expression levels of Zic2. **c**, **f**, **i** Sphere size in cell lines with different expression levels of Zic2.

This is the first analysis of the role of Zic2 in colon CSCs. To test the role of Zic2 in colon CSCs, colon cancer cells with modified Zic2 expression were cultured in anchorage-independent conditions to form tumor spheres. We observed that the size and number of spheres in Zic2-silenced colon cancer cells were remarkably decreased compared with those in control cells (Fig. 3). In addition, enhancing Zic2 expression in DLD, HCT116, and SW480 cells increased the size and number of spheres (Fig. 3). These results established that Zic2 might promote tumor formation and proliferation through the maintenance of colon CSC properties.

### Zic2 enhances Wnt signaling activity in colon cancer cells

To further reveal the underlying mechanisms by which Zic2 promotes colon tumor growth, RNA sequencing of three DLD-1-Zic2 cell lines and three DLD-1 control cell lines was performed (Supplementary Fig. 4a). Based on a *Q* value <0.05, there were 930 differentially expressed genes (DEGS), including 422 downregulated and 508 upregulated genes in ectopically expressed Zic2 clones (Supplementary Fig. 4b, c). The above results suggest that Zic2 may play both transcriptional coactivator and transcriptional repressor roles in colon cancer. Gene Ontology (GO) biological process (BP) analysis confirmed that Zic2 promotes colon tumor growth (Supplementary Fig. 4d).

**Fig. 4:**
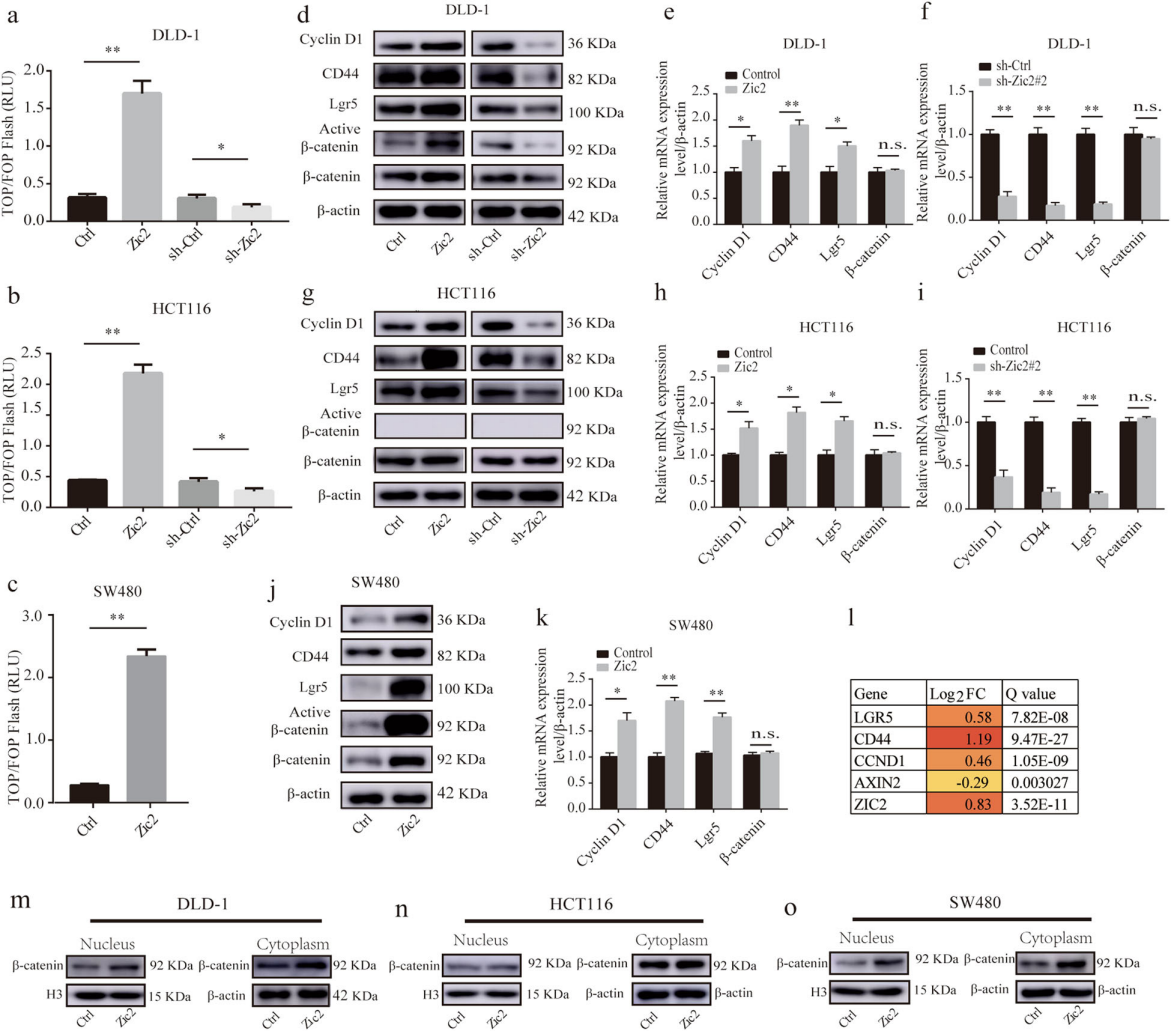
Zic2 enhances Wnt signaling activity in colon cancer cells. **a**–**c** The luciferase reporter activities in Zic2- silencing Zic2 and Zic2-overexpressing cell lines transfected with a TOP/FOP-Flash reporter plasmid. **d**, **e**, **f**, **g**, **h**, **j**, **k** The expression levels of Cyclin D1, CD44, Lgr5, active β-catenin (Ser 45), and β-catenin were detected in silenced Zic2- silencing and Zic2-overexpressing cell lines by west blotting (**d**, **g**, **j**) and qRT-PCR (**e**, **f**, **h**, **i**, **k**, **l**). **l** mRNA expression changes of Cyclin D1, CD44, Lgr5, axin2 in DLD-1-Zic2 cells and control DLD-1 cells are analyzed by RNA-seq and presented in the color-coded format. The fold changes were log2 normalized. **m**–**o** β-catenin co-immunoprecipitation with Zic2 in cell lines by western blot. All data are the mean ± SD of three independent experiments. *P < 0.05, **P < 0.01.

A recent study indicated that Zic2 can render colon cancer cells more resistant to low-glucose-induced apoptosis by activating Wnt signaling^31^. Our results demonstrate that Zic2 promotes colon cancer proliferation and CSC properties. Wnt signaling is strongly linked to tumor proliferation and CSC properties^2,3^. Therefore, we hypothesized that Wnt signaling might be involved in Zic2-mediated colon cancer growth promotion. The TOP/FOP-Flash reporter assay further verified our hypothesis (Fig. 4a–c). Ectopic expression of Zic2 in DLD-1 cells, as well as in HCT116 and SW480 cells, consistently increased luciferase activity, while Zic2 knockdown in DLD-1 and HCT116 cells decreased luciferase activity (all *P* < 0.05).

Given that Zic2 mediates the cell cycle and CSC properties, cell cycle- and CSC-related proteins were assessed in our research. We found that Zic2 initiated the transcription of canonical downstream target genes of the Wnt pathway, such as cyclin D1, CD44, and Lgr5^15,16^. In response to ectopic expression of Zic2, the expression of cyclin D1, CD44, and Lgr5 in DLD-1, HCT116, and SW480 cells was remarkably elevated at both the protein and mRNA levels (Fig. 4d–k). Correspondingly, Zic2 knockdown in DLD-1 and HCT116 cells markedly decreased the levels of cyclin D1, CD44, and Lgr5. Bioinformatic analysis via the GEPIA database (Supplementary Fig. 5a-c) and with our RNA sequencing results (Fig. 4l) further supported that Zic2 promotes the transcription of cyclin D1, CD44, and Lgr5.

In both cells with ectopic expression of Zic2 and cells with knockdown of Zic2, the transcript levels of β-catenin did not change. Moreover, the protein levels of total β-catenin and active β-catenin (Ser45) were elevated in response to ectopic expression of Zic2 in the DLD-1 and SW480 cell lines. Intriguingly, the levels of total β-catenin were not changed in the HCT116-Zic2 cell line. In addition, barely any active β-catenin protein could be detected in HCT116 cells due to the Ser45 mutation in this cell line^20^. We also found that the nuclear localization of β-catenin was unchanged in the HCT116-Zic2 cell line, but it was markedly increased in response to ectopic expression of Zic2 in the DLD-1 and SW480 cell lines (Fig. 4d, j). Taken together, the data show that Zic2 enhances Wnt signaling activity in colon cancer.

### Zic2 activates Wnt signaling in colon cancer cells by interacting with β-catenin and repressing Axin2

**Fig. 5:**
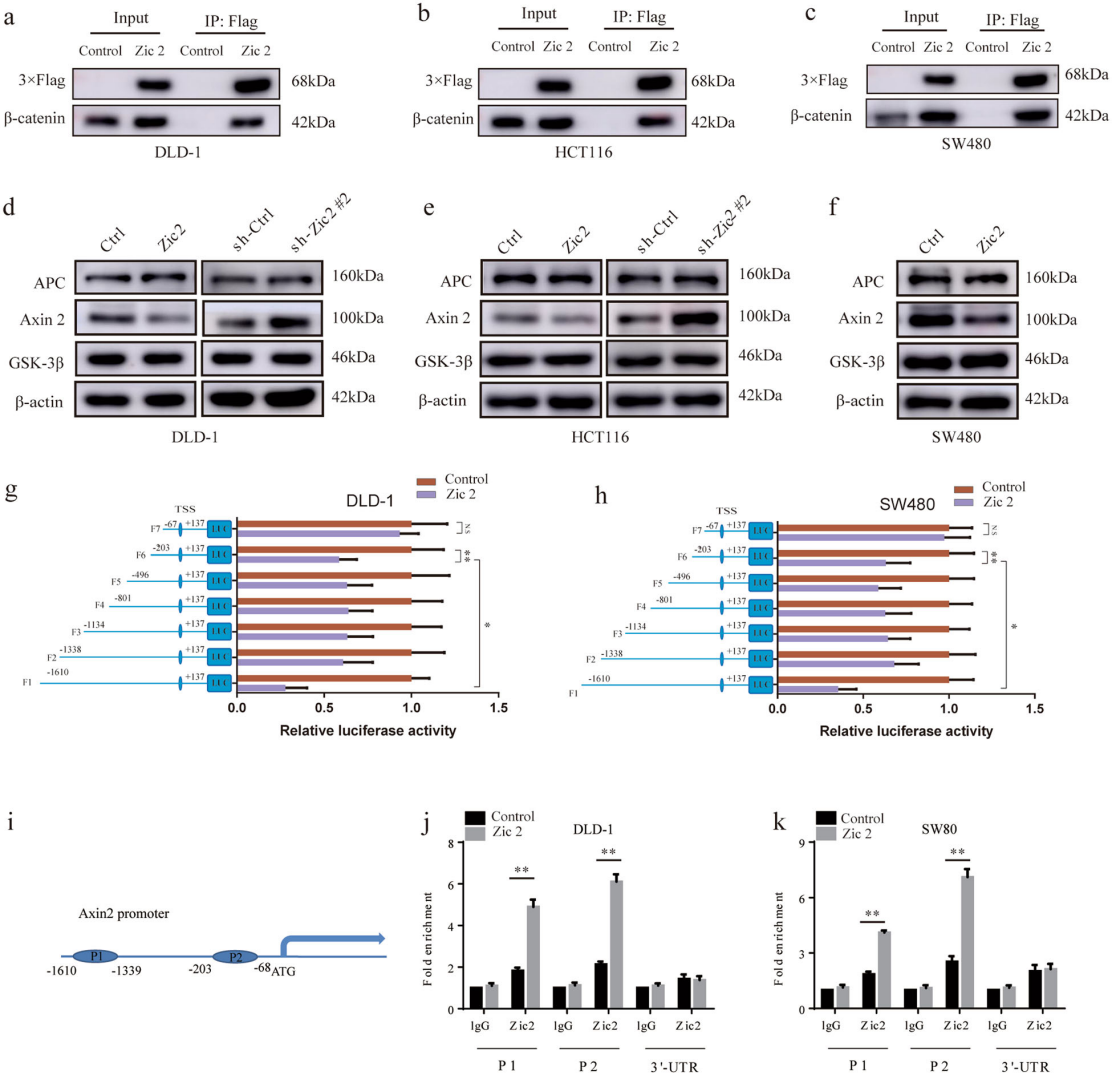
Zic2 activates Wnt signaling in colon cancer cells by interaction with β-catenin and trans-repression of Axin2. **a**–**c** Validation of β-catenin co-immunoprecipitation with Zic2 by western blot. **d**–**f** The protein levels of APC, Axin2, and GSK-3β were detected in silenced Zic2- silencing and Zic2-overexpressing cell lines by west blotting. **g**, **h** Fragments of the Axin2 promoter were constructed to generate promoter reporter. The luciferase activity relative to Renilla control was measured in Zic2-overexpressing DLD-1 or SW480 cells and their scramble infectants. **i** A schematic diagram of the two binding regions of Axin2 promoter by Zic2. **j**, **k** The qChIP assay was shown in Zic2-overexpressing DLD-1 or SW480 cells and heir scramble infectants and Immunoprecipitation by Zic2 antibody and IgG antibody. All data are presented as the mean ± SD from three independent experiments. *P < 0.05, **P < 0.01.

GO molecular function analysis suggested that Zic2 might execute its biological function in colon cancer via DNA/protein binding and transcription regulator activity (Supplementary Fig. 4e). We first demonstrated that Zic2 interacted with β-catenin in DLD-1, SW480, and HCT116 cells to activate Wnt signaling (Fig. 5a–c). Because of the differences in β-catenin levels in colon cancer cells with modified Zic2 expression, especially the peculiar unchanged β-catenin levels in HCT116 cells, we hypothesized that disruption of the β-catenin destruction complex might be essential for the enhanced Wnt signaling activity induced by Zic2 in colon cancer. The results of qPCR (Supplementary Fig. 6a–e) and RNA sequencing (Fig. 4l) analysis showed that the transcript levels of Axin2 were remarkedly decreased in response to ectopic expression of Zic2. Western blotting assays also supported that enhancing Zic2 expression decreased Axin2 protein levels (Fig. 5d, e). Correspondingly, depletion of Zic2 reversed these changes (Fig. 5d–f). Collectively, these results indicate that Zic2 exerts multilevel regulation on Wnt signaling through interaction with β-catenin and repression of Axin2 in colon cancer.

We further explored whether Zic2 activates the Wnt/β-catenin pathway via transcriptional activation of Axin2. First, a full-length Axin2 promoter (from −1610 to +137 bp) reporter construct and three other truncated constructs were constructed and transfected into DLD-Zic2 and SW480-Zic2 cells and control cells (Fig. 5g, h). The luciferase activity of the Axin2 promoter was detected by dual-luciferase reporter assay. Ectopic expression of Zic2 led to decreased luciferase activity of the full-length fragments, while cells containing fragments with truncated fragments affecting residues from −1610 to −1339 bp (P1) and from −203 to −68 (P2) showed normal luciferase activity, suggesting that Zic2 could repress Axin2 expression by binding to the P1 and P2 fragments of the Axin2 promoter (Fig. 5i). Next, we attempted to confirm whether Zic2 binds to a unique site within the Axin2 promoter in vivo by using a qChIP assay. Two pairs of primers were designed to amplify the four P1 and P2 fragments of the Axin2 promoter region. The results showed that ectopic expression of Zic2 enhanced the binding of Zic2 to the P1 and P2 fragments (Fig. 5j, k). All of these results indicate that Zic2 could bind to specific areas of the Axin2 promoter and transcriptionally activate Axin2 in colon cancer cells.

**Fig. 6:**
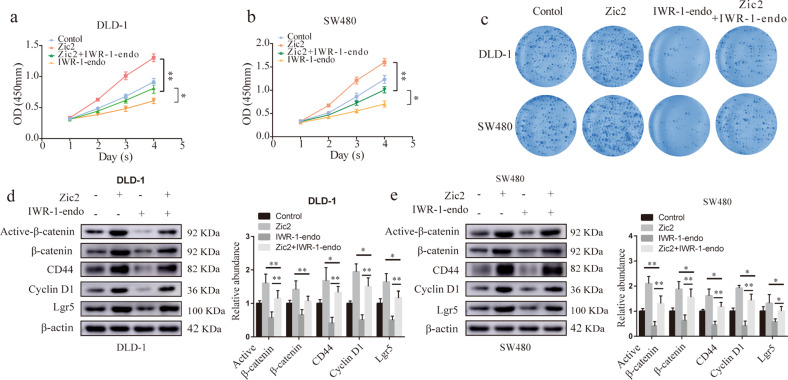
Zic2 downstream factor Axin2 contributes to colon cancer cell growth and Wnt/β-catenin activation. **a**–**c** CCK8 proliferation assay (**a**, **b**) and colony formation (**c**) were assessed in cell lines transfected with Zic2 or the vector treated with IWR-1-endo. **d**, **e** Western blotting bands for Cyclin D1, CD44, Lgr5, active β-catenin (Ser 45), and β-catenin were detected in DLD-1 (**c**) and SW480 (**d**) transfected with Zic2 or the vector treated with IWR-1-endo. All data are presented as the mean ± SD from three independent experiments. *P < 0.05, **P < 0.01.

To further determine whether Axin2 is involved in Zic2-mediated Wnt signaling activation, we stabilized Axin2 using IWR-1-endo and further investigated its impact on the functions of Zic2 (Fig. 6a–c). Treating DLD-1-Zic2 and SW480-Zic2 cells with IWR-1-endo remarkably attenuated cell viability and colony formation in vitro. Furthermore, following the decrease in Axin2 levels induced by Zic2, Wnt/β-catenin signaling was recovered, as visualized by elevated levels of β-catenin, cyclin D1, CD44, and Lgr5 (Fig. 6d, e). Taken together, these results led us to conclude that Zic2 promotes colon cancer growth by activating Wnt signaling via interaction with β-catenin and repression of Axin2.

### Correlation between Zic2 and Wnt signaling in vivo

**Fig. 7:**
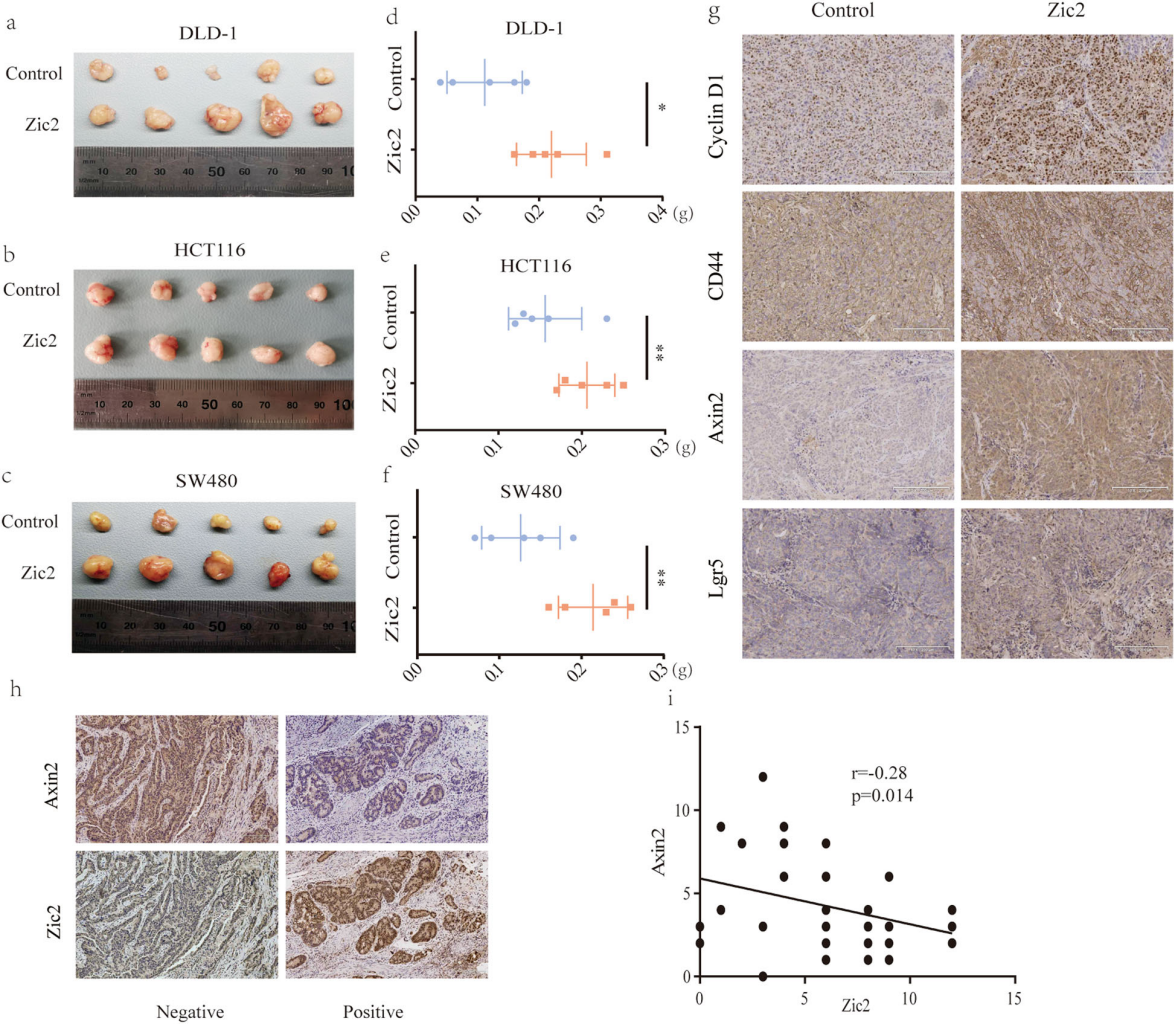
Correlation between Zic2 and Wnt signaling in vivo. **a**–**c** Images of xenografts 21 days after inoculation of cell liners transfected with Zic2 or the vector. **d**–**f** Weight of xenografts at 21 days after inoculation of cell liners transfected with Zic2 or the vector. **g** Immunohistochemistry for CD44, Cyclin D1, Axin2, and Lgr5 in DLD-1 transfected with Zic2 or the vector. **h** Representative images of immunohistochemical staining showing the Zic2, Axin2 expression in CRC tissues. **i** Correlation between IHC staining scores of the Zic2 and Axin2 in 42 colon cancer tissues. All data are presented as the mean ± SD from three independent experiments. *P < 0.05, **P < 0.01.

To validate the function of Zic2 in vivo, Zic2-overexpressing colon cancer cells and negative control cells were subcutaneously injected into nude mice. Three weeks later, we measured the weight of xenograft tumors and found that ectopic expression of Zic2 significantly augmented the weight of xenograft tumors (Fig. 7a–f). By IHC analysis of the xenograft tumors, we found that Zic2 was positively associated with the expression of cyclin D1, CD44, and Lgr5, and negatively associated with the expression of Axin2 (Fig. 7g). The inverse correlation between Zic2 and Axin2 was further validated by IHC analysis of human colon cancer samples (Fig. 7h, i). Our data confirmed that Zic2 activated Wnt signaling by repressing Axin2 in vivo.

## Discussion

Zic2 is upregulated and correlates with poor survival in various cancers, such as hepatocellular carcinoma^30^, clear cell renal cell carcinoma^41^, oral squamous cell carcinoma^42^, and epithelial ovarian cancer^43^. In our study, a similar conclusion was drawn in colon cancer, and this is the first analysis of the relationship between Zic2 and prognosis using human colon cancer samples. However, Zic2 is barely expressed in breast cancer, in which high Zic2 expression predicts better survival. The reason for its positive relationship with prognosis in breast cancer is because in this cancer, Zic2 directly represses STAT3 transcription^29^. However, in most cancers, Zic2 acts as a transcriptional activator promoting tumorigenesis^21,30,31^. Intriguingly, we demonstrated for the first time that Zic2 acts as both a transcriptional coactivator and transcriptional repressor in colon cancer. Zic2 directly represses Axin2 transcription, which results in the disruption of the β-catenin destruction complex and the subsequent accumulation of active β-catenin in SW480 and DLD-1 cells. More intriguingly, ectopic expression of Zic2 in HCT116 cells did not change the level of active β-catenin, which might be due to the in-frame deletion at Ser45 in HCT116 cells^20^. Given that Zic2 activated Wnt/β-catenin signaling in HCT116 cells, we speculated that there exists another mechanism of Zic2-mediated Wnt signaling activation. As expected, we confirmed that Zic2 interacts with β-catenin in SW480 and DLD-1 cell lines besides the HCT116 cell line^31^. Zic2 might enhance Wnt/β-catenin signaling in colon cancer via chromatin remodeling^21,44,45^. Collectively, in addition to directly repressing Axin2 transcription, Zic2 also interacts with β-catenin to activate Wnt signaling in colon cancer. Our study revealed multilevel regulation of Wnt signaling by Zic2 in colon cancer.

A previous study reported that Zic2 enhances Wnt signaling and plays crucial roles in tumorigenesis, and the same was found in our study^31^. However, limited downstream target genes affected by the Zic2-Wnt/β-catenin axis have been identified, with only Glut1 being reported so far^31^. In the present study, we identified that Zic2 enhances Wnt signaling and initiates the transcription of several novel downstream target genes, including cyclin D1, CD44, and Lgr5, which promote proliferation and stemness in colon cancer cells.

A role of Zic2 in proliferation has not been conclusively demonstrated. Zic2 promotes proliferation in various cancers^30,31^ but not in breast cancer^29^. Zic2^−/−^ mice display a normal rate of proliferation^46^. Interestingly, Ishiguro et al. reported that phosphorylation of Zic2 at Ser200 decreases the interaction between Zic2 and RNA helicase in mice, which can suppress the transcriptional activation potential of Zic2^47^. However, posttranslational modification and mutation of Zic2 cannot be excluded as reasons for our current findings, and the influence of these factors remains to be further evaluated. The discrepancy in its effects in different cancers makes it difficult to define the role of Zic2 in proliferation. In our study, ectopic expression of Zic2 promoted colon cancer proliferation by activating cyclin D1 transcription, in addition to cell cycle transition from G0/G1 to S phase in vitro. Chandrasekaran et al. reported that silencing Zic2 expression in benign prostate epithelial cells inhibited anchorage-independent spheroid and tumor formation by repressing CD44, Sox2, and Notch1 expression, indicating that Zic2 might be closely associated with self-renewal properties^26^. A similar conclusion was made in liver cancer stem cells^21^. Herein, we identified for the first time that Zic2 accelerates sphere formation in vitro. Zic2 upregulated CD44 and Lgr5, well-known CSC markers^14,48–52^, via Wnt/β-catenin signaling. Our study also revealed that Zic2 has effects on the maintenance of colon CSCs.

## Conclusions

In summary, we demonstrated that Zic2 is upregulated in colon cancer samples and that high Zic2 expression correlates with unfavorable survival of colon cancer patients. Our results indicate that Zic2 promotes the proliferation and tumorigenesis of colon cancer. Zic2 functions in colon cancer, at least in part, by enhancing Wnt/β-catenin signaling via collaboration with β-catenin and repressing Axin2. The above results suggest that the inhibition of Zic2 might be a potential therapeutic strategy for colon cancer patients.

## Supplementary information

Supplementary Table1-5.docx

Supplemental Figure Legend

Supplementary. Figure 1

Supplementary. Figure 2

Supplementary. Figure 3

Supplementary. Figure 4

Supplementary. Figure 5

Supplementary. Figure 6

## Data Availability

The datasets generated and/or analyzed during the current study are not publicly available but are available from the corresponding author on reasonable request.
