# Supplementary material for: Multilevel regulation of Wnt signaling by Zic2 in colon cancer due to mutation of β-catenin
Source: Cell Death Dis. 2021 Jun 7;12(6):584. doi: 10.1038/s41419-021-03863-w (PMC8184991; doi:10.1038/s41419-021-03863-w)
Supplement: Supplementary file 1 — Supplementary Table1-5.docx [file 41419_2021_3863_MOESM1_ESM.docx]

| **Suppl. Table 1 Correlation between Zic2 expression and clinicopathological characteristics in two independent cohorts of human colon cancer tissues** | | | | | | | | |
| --- | --- | --- | --- | --- | --- | --- | --- | --- |
|  | **Cohort Ⅰ** | | | | **Cohort Ⅱ** | | | |
|  | Low | High | χ2 | P | Low | High | χ2 | P |
| Age |  |  | 0.081 | 0.776 |  |  | 2.334 | 0.127 |
| ≥60 | 23 | 84 |  |  | 47 | 167 |  |  |
| ＜60 | 17 | 56 |  |  | 54 | 135 |  |  |
| Sex |  |  | 0.931 | 0.335 |  |  | 0.041 | 0.909 |
| male | 20 | 82 |  |  | 51 | 156 |  |  |
| female | 20 | 58 |  |  | 50 | 146 |  |  |
| Tumor size |  |  | 8.693 | 0.003 |  |  | 7.146 | 0.008 |
| ≥5 | 12 | 79 |  |  | 39 | 158 |  |  |
| ＜5 | 28 | 61 |  |  | 62 | 144 |  |  |
| T |  |  | 7.986 | 0.005 |  |  | 9.598 | 0.02 |
| T1+T2 | 16 | 26 |  |  | 34 | time |  |  |
| T3+T4 | 24 | 114 |  |  | 67 | 250 |  |  |
| Lymph node metastasis | |  | 3.348 | 0.067 |  |  | 0.103 | 0.748 |
| yes | 11 | 61 |  |  | 33 | 110 |  |  |
| no | 29 | 79 |  |  | 68 | 192 |  |  |
| Tumor differentiation |  |  | 0.0673 | 0.423 |  |  | 1.623 | 0.203 |
| well or moderate | 35 | 115 |  |  | 87 | 247 |  |  |
| poor | 5 | 25 |  |  | 13 | 55 |  |  |
| TNM stage |  |  | 7.642 | 0.022 |  |  | 7.753 | 0.021 |
| Ⅰ | 12 | 18 |  |  | 19 | 27 |  |  |
| Ⅱ | 17 | 64 |  |  | 48 | 165 |  |  |
| Ⅲ | 11 | 61 |  |  | 33 | 110 |  |  |

| **Suppl. Table 2 Univariate and multivariate analysis of factors associated with oversurvival in two independent cohorts of human colon cancer** | | | | | | | | |
| --- | --- | --- | --- | --- | --- | --- | --- | --- |
|  | **CohortⅠ** | | | | | | | |
|  | Univariate analysis | | | | Multivariate analysis | | | |
| Clinicopathological variables | Hazard Ratio | CI (Lower) | CI (Upper) | P-value | Hazard Ratio | CI (Lower) | CI (Upper) | P-value |
| Age (＜60 Versus ≥60) | 1.095 | 0.658 | 1.821 | 0.727 | 0.968 | 0.574 | 1.634 | 0.904 |
| Sex (Male Versus Female） | 1.108 | 0.675 | 1.818 | 0.686 | 1.008 | 0.606 | 1.676 | 0.975 |
| Tumor size(＜5cm Versus ≥5cm) | 1.306 | 0.794 | 2.148 | 0.292 | 1.124 | 0.672 | 1.88 | 0.657 |
| T stage (T1+T2 Versus T3+T4) | 1.046 | 0.577 | 1.894 | 0.883 | 0.834 | 0.452 | 1.538 | 0.56 |
| Lymph node metastasis  (No Versus Yes) | 2.407 | 1.462 | 3.961 | 0.001 | 1.766 | 1.032 | 3.021 | 0.038 |
| Tumor differentiation  (Well or moderate Versus Poor) | 2.994 | 1.755 | 5.108 | ＜0.001 | 2.308 | 1.297 | 4.108 | 0.004 |
| Zic2 (Low versus High） | 2.758 | 1.254 | 6.6069 | 0.012 | 2.317 | 1.018 | 5.273 | 0.045 |
|  | **CohortⅡ** | | | | | | | |
|  | Univariate analysis | | | | Multivariate analysis | | | |
| Clinicopathological variables | Hazard Ratio | CI (Lower) | CI (Upper) | P-value | Hazard Ratio | CI (Lower) | CI (Upper) | P-value |
| Age (＜60 Versus ≥60) | 1.201 | 0.839 | 1.72 | 0.317 | 1.047 | 0.726 | 1.512 | 0.805 |
| Sex (Male Versus Female） | 1.316 | 0.921 | 1.882 | 0.132 | 1.232 | 0.86 | 1.763 | 0.255 |
| Tumor size(＜5cm Versus ≥5cm) | 1.168 | 0.818 | 1.668 | 0.392 | 1.127 | 0.783 | 1.623 | 0.52 |
| T stage (T1+T2 Versus T3+T4) | 1.411 | 0.881 | 2.261 | 0.152 | 1.345 | 0.828 | 2.184 | 0.231 |
| Lymph node metastasis  (No Versus Yes) | 2.226 | 1.559 | 3.178 | ＜0.001 | 1.99 | 1.38 | 2.868 | ＜0.001 |
| Tumor differentiation  (Well or moderate Versus Poor) | 2.204 | 1.465 | 3.317 | ＜0.001 | 1.779 | 1.161 | 2.726 | 0.008 |
| Zic2 (Low versus High） | 1.983 | 1.248 | 3.149 | 0.004 | 1.631 | 1.017 | 2.615 | 0.043 |

| **Suppl. Table 3 shRNA target sequences** | | |
| --- | --- | --- |
| **Name** | **Source** | **Target Sequences** |
| shZIC2#1 |  | TCTCCATGCCCACGTTCTT |
| shZIC2#2 |  | GCGACAGGAAGAAGCACAT |

| **Suppl. Table 4 Primer sequence** | | | |
| --- | --- | --- | --- |
| **Gene Name** | **Full Name** | **Gene ID** | **Sequences** |
| **RT-PCR** |  |  |  |
| ZIC2 | Zic family member 2 | 7546 | F:ACAAAAGGACCCACACAGGG |
|  |  |  | R:CATGATCACAAGGTGCCCTC |
| β-actin | actin beta | 60 | F:CTCCATCCTGGCCTCGCTGT |
|  |  |  | R:GCTGTCACCTTCACCGTTCC |
| APC | APC regulator of WNT signaling pathway | 324 | F:AAAATGTCCCTCCGTTCTTATGG |
|  |  |  | R:CTGAAGTTGAGCGTAATACCAGT |
| AXIN2 | axin 2 | 8313 | F:AGCCAAAGCGATCTACAAAAGG |
|  |  |  | R:AAGTCAAAAACATCTGGTAGGCA |
| GSK-3β | glycogen synthase kinase 3 beta | 2932 | F:GGCAGCATGAAAGTTAGCAGA |
|  |  |  | R:GGCGACCAGTTCTCCTGAATC |
| CCND1 | cyclin D1 | 595 | F:GCTGCGAAGTGGAAACCATC |
|  |  |  | R:CCTCCTTCTGCACACATTTGAA |
| CD44 | CD44 molecule | 960 | F: CCCTGCTACCAGAGACCAAGAC |
|  |  |  | R: GCAGGTTCCTTGTCTCATCAGC |
| beta-Catenin | catenin beta 1 | 1499 | F:CATTACAACTCTCCACAACC |
|  |  |  | R:CAGATAGCACCTTCAGCAC |
| CK1 | casein kinase 1 alpha 1 | 1452 | F:TAACAAGATGGCGTCGTCCG |
|  |  |  | R:TGCTCTCGTACAGCAACTGG |
| AXIN1 | axin 1 | 8312 | F:GTGCCCCTACCTCACATTCC |
|  |  |  | R:GTGCCCCTACCTCACATTCC |
| **Luciferase Assays** |  |  |  |
| F1(-1610bp-+137bp) | | | F:CCGGCCCCTTTTGCGGAGAG |
|  |  |  | R:GTGACCTGGCCCTTGCCCAC |
| F2(-1338bp-+137bp) | | | F:TCCAGGTAACGGGGCCGCTC |
|  |  |  | R:GTGACCTGGCCCTTGCCCAC |
| F3(-1134bp-+137bp) | | | F:TTCCGTGCTAGGGAAAGCAGC |
|  |  |  | R:GTGACCTGGCCCTTGCCCAC |
| F4(-801bp-+137bp) | | | F:TCAAATCTGAGTTGGGGAGGGGGT |
|  |  |  | R:GTGACCTGGCCCTTGCCCAC |
| F5(-496bp-+137bp) | | | F:AAGGGAGGGAAGGCGAGGCG |
|  |  |  | R:GTGACCTGGCCCTTGCCCA |
| F6(-203bp-+137bp) | | | F:TCTTGCTTTGATGTTGGGTAGATCTGG |
|  |  |  | R:GTGACCTGGCCCTTGCCCAC |
| F7(-67bp-+137bp) | | | F:ACGCCGATTGCTGAGAGGAACT |
|  |  |  | R:GTGACCTGGCCCTTGCCCAC |
| **ChIP-qPCR** |  |  |  |
| P1(-1610bp--1339bp) | | | F:GTGTCCCTCGCGTCCCTCCT |
|  |  |  | R:GAACCCCGGGACACGCCAG |
| P2(-203bp--68bp) | | | F:TCTCTCTCCCCACCTCCCCC |
|  |  |  | R:GAAGCTGCTGCTGGGGTCCG |
| 3'-UTR | | | F:GCCCAAGCCCGTCTCTGACC |
|  |  |  | R:ATCCCCTGGTCCCCTGCACC |

| **Suppl. Table 5 The details of antibodies** | | | |
| --- | --- | --- | --- |
| Name | Company | Catalog No. | Application(s) |
| Zic2 | Abcam | ab150404 | WB, IP, COIP, CHIP, IF |
| Zic2 | Avia systems biology | ARP-35821_P050 | IHC |
| β-catenin | Abcam | #8480 | WB |
| Active β-catenin^Ser45^ | CST | #19807 | WB |
| Cyclin D1 | CST | #2978 | WB, IHC |
| CD44 | Abcam | ab189524 | WB, IHC |
| Lgr5 | Abcam | ab75850 | WB, IHC |
| Axin2 | Abcam | ab109307 | WB, IHC |
| APC | Abcam | ab40778 | WB |
| GSK-3β | CST | #12456 | WB |
| DDDDK tag | Proteintech | 20543-1-AP | WB |
| Histone H3 | Abcam | ab32356 | WB |
| β-actin | Abcam | ab8227 | WB |
