# Supplementary material for: Multilevel regulation of Wnt signaling by Zic2 in colon cancer due to mutation of β-catenin
Source: Cell Death Dis. 2021 Jun 7;12(6):584. doi: 10.1038/s41419-021-03863-w (PMC8184991; doi:10.1038/s41419-021-03863-w)
Supplement: Supplementary file 2 — Supplemental Figure Legend [file 41419_2021_3863_MOESM2_ESM.docx]

**Supplementary Figure. 1 Identification of the distribution and expression of Zic2 in cell lines.** **a** the distribution (green) of Zic2 in DLD-1. **b, c** the protein (b) and mRNA (c) levels of Zic2 protein in cell lines. c the mRNA expression of Zic2 in cell lines. **d, e, f** the protein (d, e) and mRNA (f) levels of Zic2 protein in cell lines transfected with sh-Zic2 or the vector. **g, h, i, j** the protein (g, h, i) and mRNA (j) levels of Zic2 protein in cell lines transfected with Zic2 or the vector

**Supplementary Figure.** 2 **Cell cycle distribution in vivo according to Zic2 expression**

**Supplementary Figure. 3 Correlation between Zic2 expression and apoptosis in vivo**

**Supplementary Figure. 4 Bioinformatic analysis of RNA-seq of three DLD-1-Zic2 cell lines and three DLD-1 negative control cell lines a** Pheatmap of DLD-1 control and DLD-1-Zic2 **b** Volcano Plot of DLD-1 control and DLD-Zic2 **c** Number of DEGs (Q value ﹤0.05) between DLD-1 control and DLD-1-Zic2 **e**, **f** Gene Ontology (GO) biological process analysis and molecular function analysis according to DEGs between DLD-1 control and DLD-1-Zic2

**Supplementary Figure. 5 Correlation analysis of Zic2 and cyclin D1(a), CD44 (b), and Lgr5 (c) in GEPIA database**

**Supplementary Figure. 6 mRNA expression levels of APC, GSk-3β, Axin1, Axin2, CK1 according to Zic2 mRNA expression.**
