# Supplementary figures and images for: Multilevel regulation of Wnt signaling by Zic2 in colon cancer due to mutation of β-catenin
Source: Cell Death Dis. 2021 Jun 7;12(6):584. doi: 10.1038/s41419-021-03863-w (PMC8184991; doi:10.1038/s41419-021-03863-w)

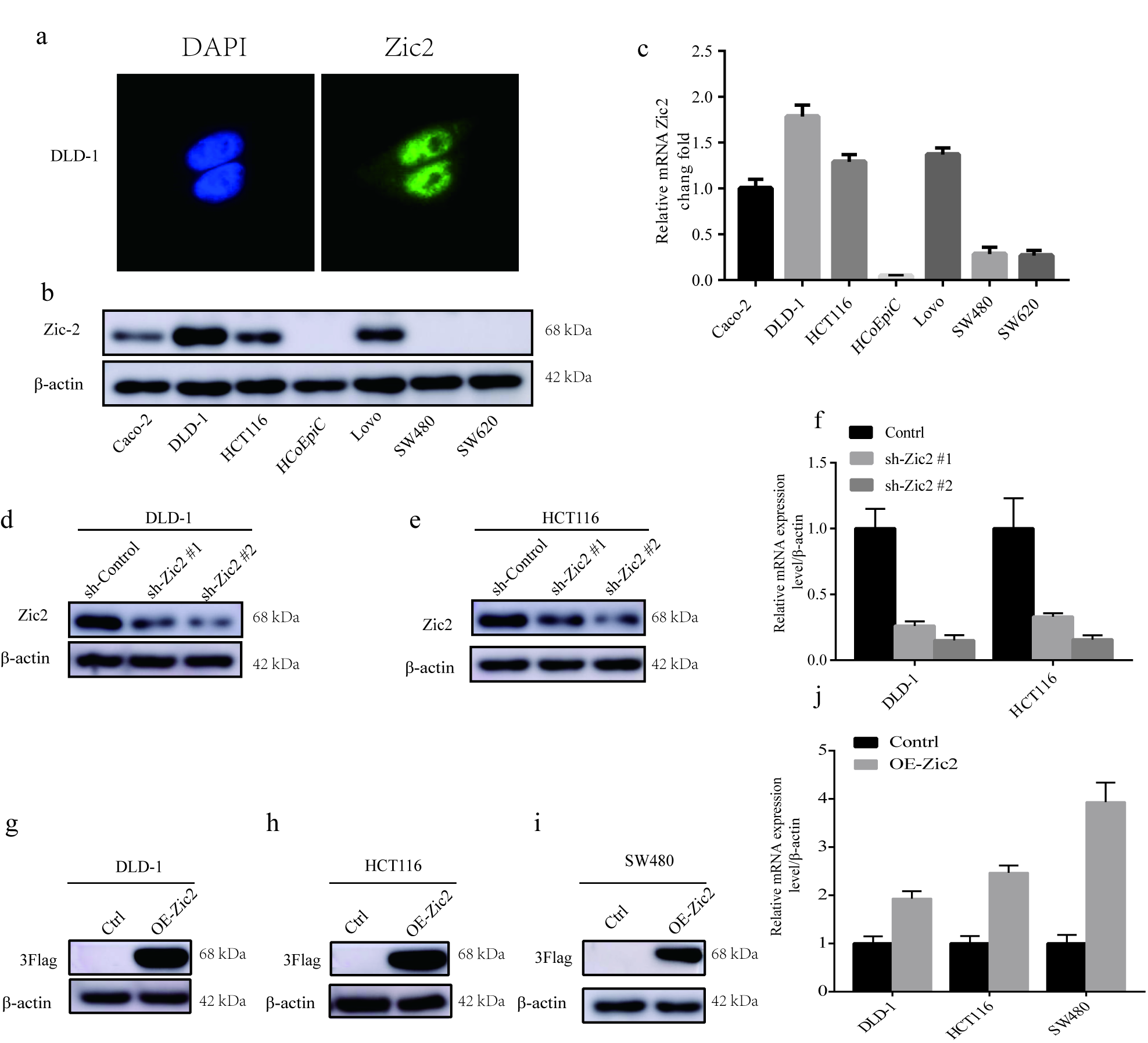

Supplement: Supplementary file 3 — Supplementary. Figure 1 [file 41419_2021_3863_MOESM3_ESM.tif]

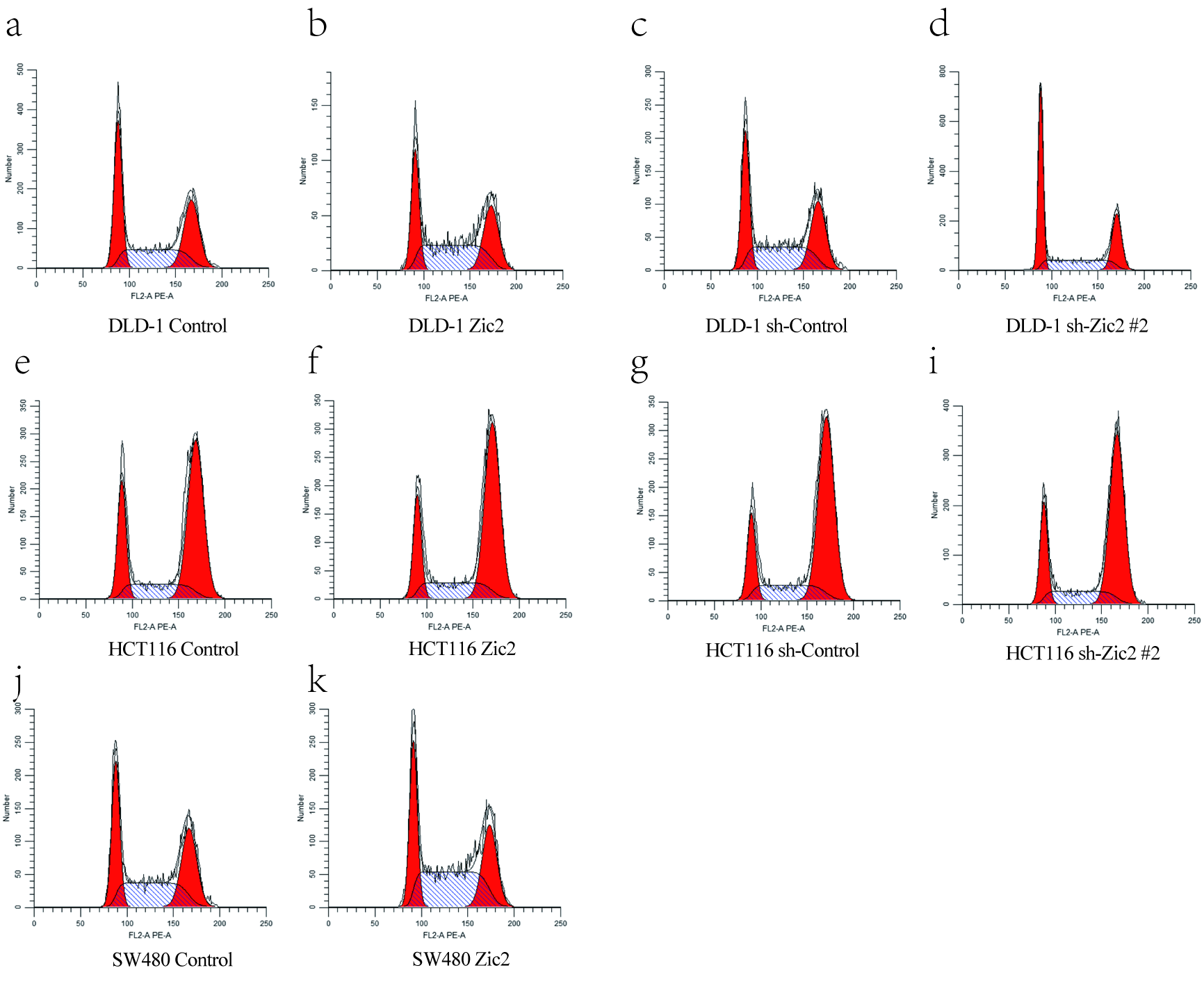

Supplement: Supplementary file 4 — Supplementary. Figure 2 [file 41419_2021_3863_MOESM4_ESM.tif]

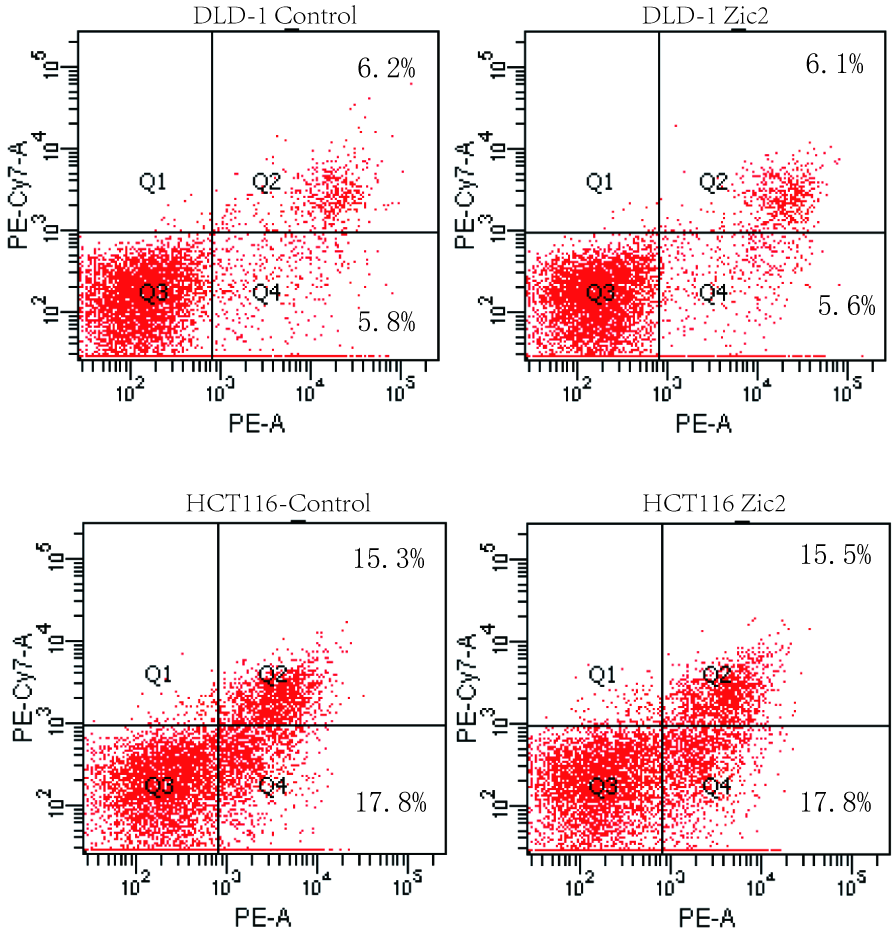

Supplement: Supplementary file 5 — Supplementary. Figure 3 [file 41419_2021_3863_MOESM5_ESM.tif]

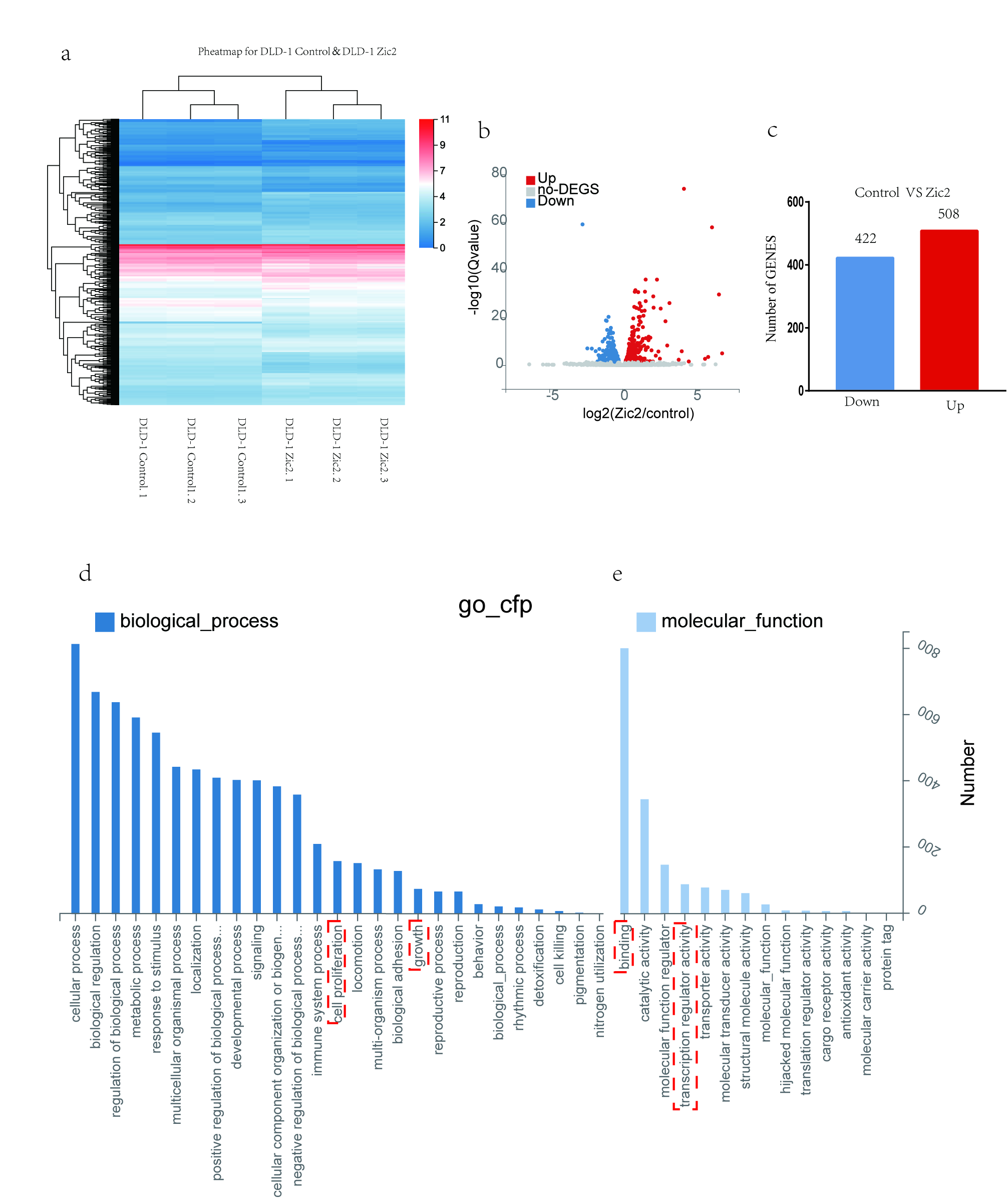

Supplement: Supplementary file 6 — Supplementary. Figure 4 [file 41419_2021_3863_MOESM6_ESM.tif]

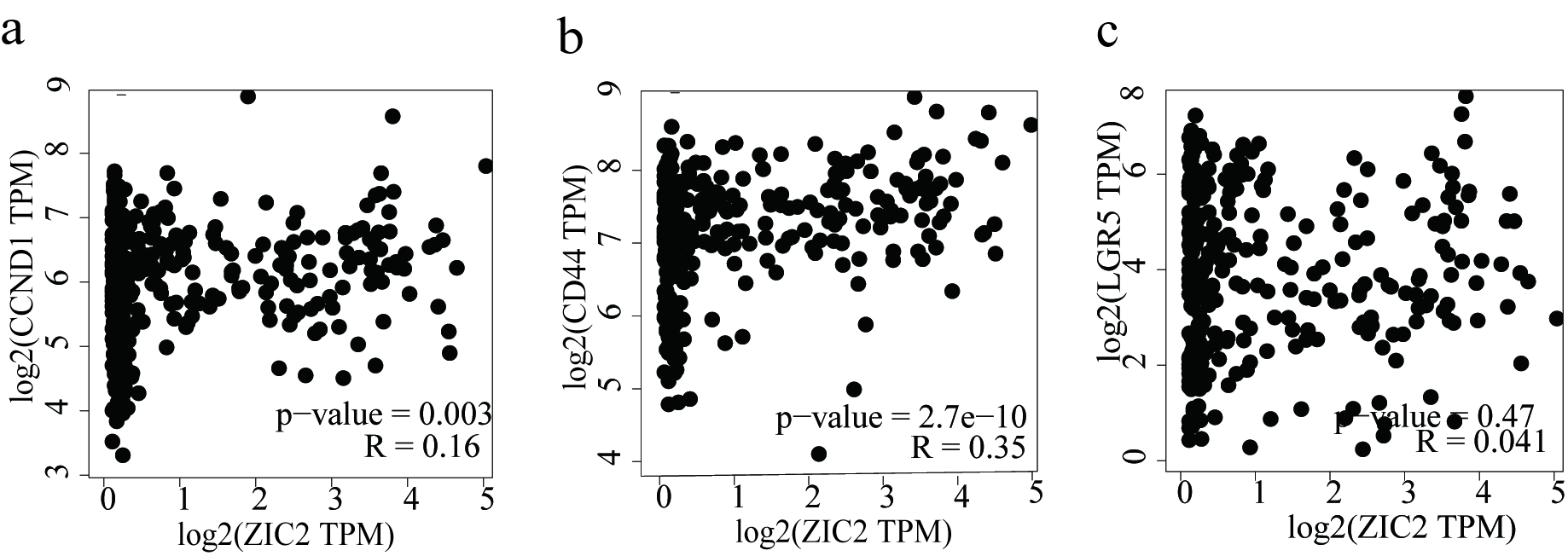

Supplement: Supplementary file 7 — Supplementary. Figure 5 [file 41419_2021_3863_MOESM7_ESM.tif]

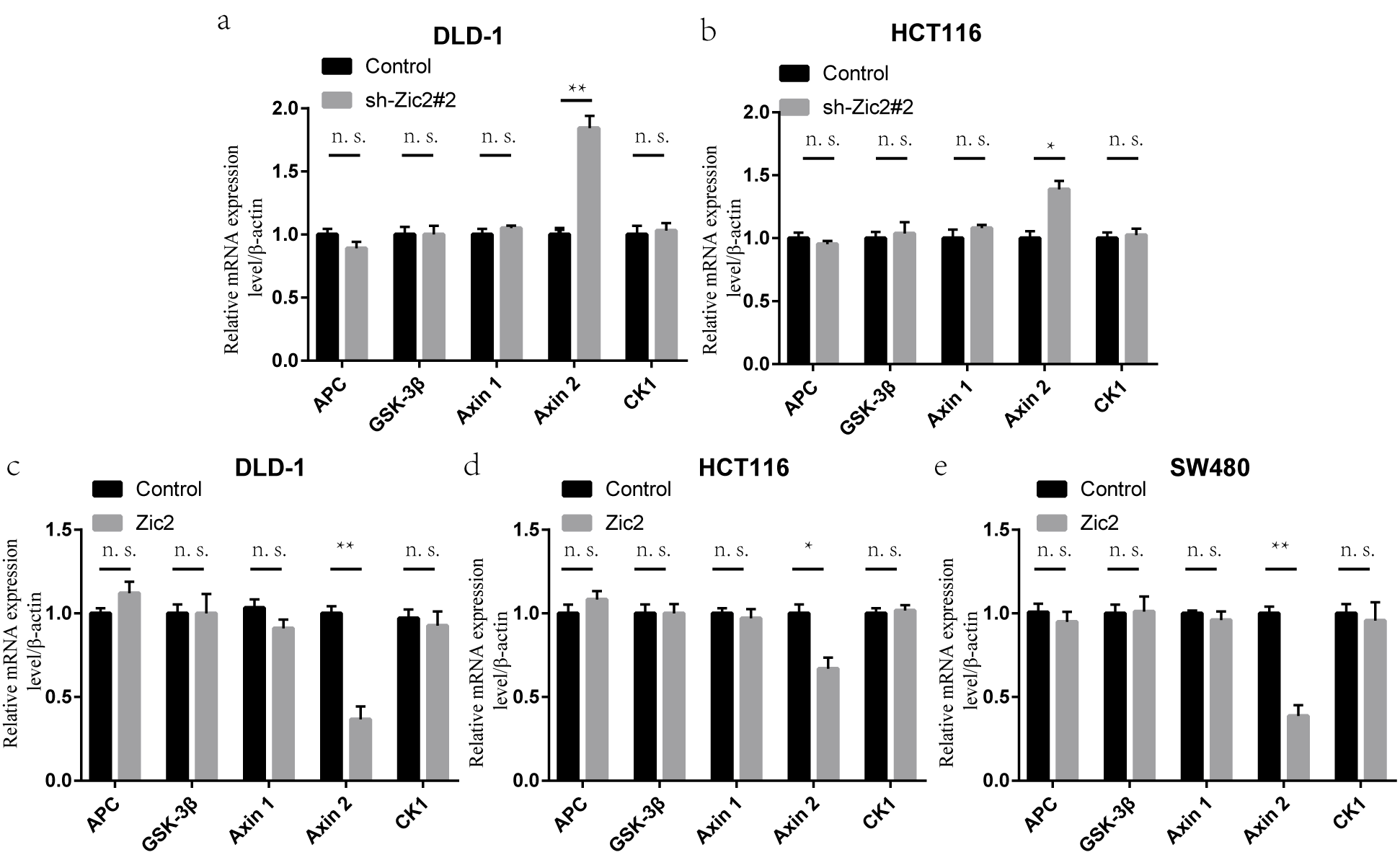

Supplement: Supplementary file 8 — Supplementary. Figure 6 [file 41419_2021_3863_MOESM8_ESM.tif]
